# Supplementary material for: Andreev reflection of fractional quantum Hall quasiparticles
Source: Nat Commun. 2021 May 14;12:2794. doi: 10.1038/s41467-021-23160-6 (PMC8121820; doi:10.1038/s41467-021-23160-6)
Supplement: Supplementary file 1 — Supplementary Information [file 41467_2021_23160_MOESM1_ESM.pdf]

Supplementary Information for
"Andreev reflection of fractional quantum Hall quasiparticles"

M. Hashisaka[1,2], T. Jonckheere[3], T. Akiho[1], S. Sasaki[1], J. Rech[3], T. Martin[3] and K. Muraki[1]

[1]NTT Basic Research Laboratories, NTT Corporation, 3-1 Morinosato-Wakamiya, Atsugi, Kanagawa 243-0198, Japan
[2]JST, PRESTO, 4-1-8 Honcho, Kawaguchi, Saitama 332-0012, Japan
[3]Aix Marseille Univ, Université de Toulon, CNRS, CPT, Marseille, France

Supplementary Note 1: Landauer-Büttiker edge transport picture

Supplementary Fig. 1 is a schematic of the experimental setup showing the whole of the Hall-bar device, where the blue and red arrows show the $\nu = 1$ and 1/3 edge channels, respectively. When the counter-propagating $\nu = 1$ and 1/3 channels are fully equilibrated at the wide junction across the Hall bar, a chiral one-dimensional channel of conductance $2e^2/3h$ is formed at the junction, as shown by a black arrow. The channels incoming to the narrow junction do not experience equilibration with any other channels before impinging on it, because both bulk $\nu = 1$ and 1/3 states are insulating (incompressible). Therefore, the incoming voltages V_1 and V_3 correspond to the voltages of the electrodes at their upstream. Actually, we observe $V_1 = V_{\text{in}}$ and $V_3 = 0$ over the entire range of V_S, as shown in Figs. 2b and 2c. When the narrow junction has conductance g, the transmitted current I is given by $I = g(V_1 - V_3) = gV_{\text{in}}$; hence, we find $g = G$. This justifies the picture in Fig. 1c. Resultantly, the outgoing voltages V_2 and V_4 are expressed as $V_2 = V_{\text{in}} - I \times (3h/e^2) = [1 - G(e^2/3h)^{-1}]V_{\text{in}}$ and $V_4 = I \times (h/e^2) = G(e^2/h)^{-1}V_{\text{in}}$, as described in the main text.

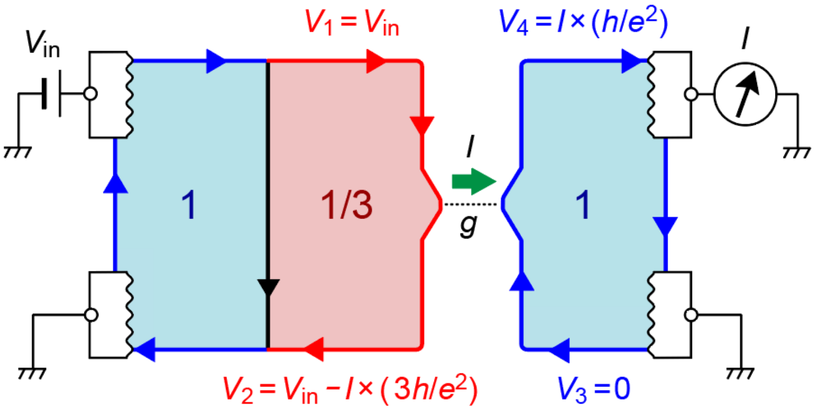

Supplementary Figure 1. Schematic of experimental setup.

Supplementary Note 2: Longitudinal and vertical resistances of the two-dimensional electron system

Supplementary Fig. 2a shows the longitudinal (R_{xx}) and vertical (R_{xy}) resistances of the bulk two-dimensional electron system (2DES) as a function of the perpendicular magnetic field B at the back-gate voltage V_{BG} = 1.29 V. Supplementary Fig. 2b shows a colour plot of R_{xx} in the V_{BG}-B plane. The experimental results presented in the main text were obtained at B = 9.0 T and V_{BG} = 1.29 V ($\nu \cong$ 1), indicated by the dotted line in Supplementary Fig. 2a and the white circle in Supplementary Fig. 2b.

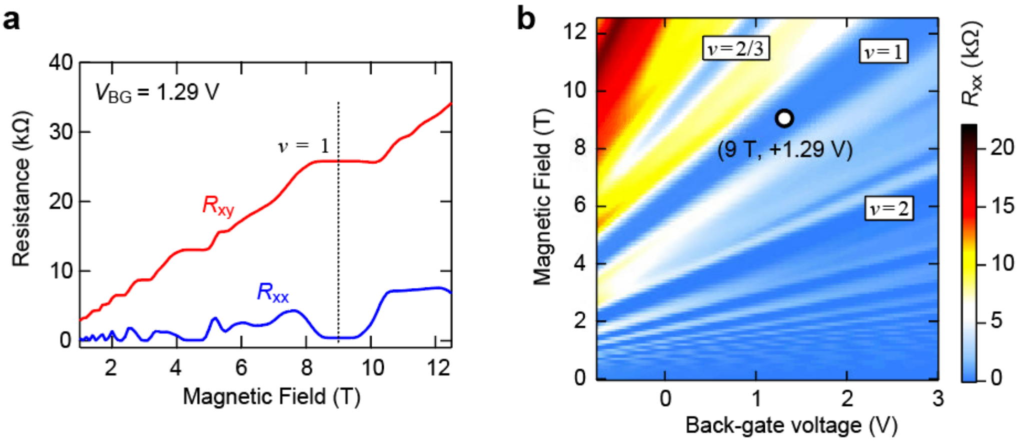

Supplementary Figure 2. a, Magnetic field dependence of R_{xx} and R_{xy}. **b,** Colour plot of R_{xx} as a function of V_{BG} and B.

Supplementary Note 3: Asymmetric split-gate biasing

Supplementary Figs. 3a and 3b show the colour plots of G measured as a function of the gate voltages V_{S1} and V_{S2} independently applied to the upper and lower split-gate electrodes (see Supplementary Fig. 3d). The two graphs show the same experimental results in different ranges: $0.9 \times e^2/3h \leq G \leq 1.1 \times e^2/3h$ in Supplementary Fig. 3a; $0 \leq G \leq 1.25 \times e^2/3h$ in Supplementary Fig. 3b. Whereas $G = e^2/3h$ at $V_{S1} > -0.55$ V and/or $V_{S2} > -0.55$ V, G deviates from $e^2/3h$ when both V_{S1} and V_{S2} are below –0.55 V, because the 2DESs under the split-gate metals are depleted near –0.55 V. In this area, we observe the conductance oscillations with $G > e^2/3h$ (shown in red in Supplementary Fig. 3a) that are the signatures of several Andreev processes. Some peaks and dips in the conductance oscillations are likely to appear parallel to either V_{S1} or V_{S2} axis [e.g. along the line (i) in Supplementary Fig. 3b], suggesting that the number of scatterers in the junction decreases one by one with these gate voltages. Supplementary Fig. 3c shows G traces as a function of the split-gate voltages swept along the four lines in Supplementary Fig. 3b. We observe a variety of conductance oscillations that reflect the positions of scatterers in the 1/3-1 junction.

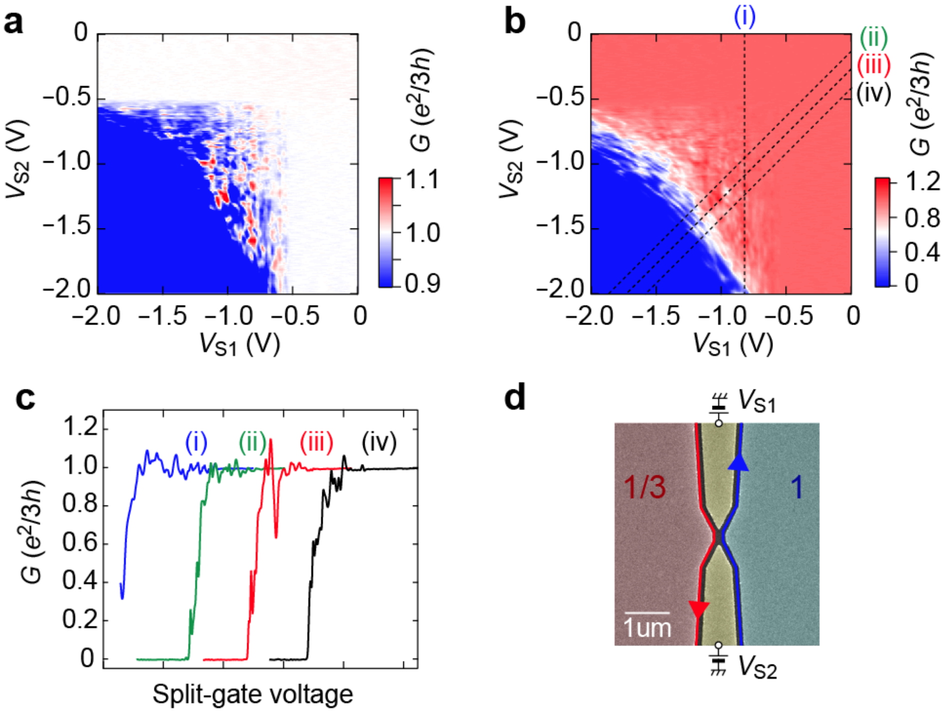

Supplementary Figure 3. a,b, Colour plots of G as a function of V_{S1} and V_{S2} in different G ranges: $0.8 \times e^2/3h \le G \le 1.2 \times e^2/3h$ in **a** and $0 \le G \le 1.25 \times e^2/3h$ in **b**. **c,** Pinch-off characteristics of G obtained by the split-gate voltage sweeps along the dashed lines in **b** (horizontally shifted for clarity). **d,** Gate voltages V_{S1} and V_{S2} applied to the split gate.

Supplementary Note 4: Resonant tunnelling through discrete levels

While our experimental results can be well understood with the multiple-scatterer model, here we complement our discussion by excluding the possibility that the conductance oscillations originate from resonant tunnelling through unintentionally formed discrete levels. Such discrete levels could form when puddles of different filling factors exist near the junction[1]. First of all, the presence of a discrete level by itself cannot induce $G > e^2/3h$, because in the absence of the Andreev process the conductance is upper-limited to $e^2/3h$ of the $\nu = 1/3$ region. Second, the increase in the oscillation amplitude with decreasing V_S (Fig. 2a) is inconsistent with the V_S dependence of the tunnel barrier height that increases with decreasing V_S. Third, when we independently sweep the split-gate voltages V_{S1} and V_{S2} (Supplementary Fig. 3d), no features of resonant tunnelling are seen in the region of $G > 0.8 \times e^2/3h$ (Supplementary Fig. 3a); the conductance peaks and dips are likely to appear parallel to either V_{S1} or V_{S2}. This observation indicates that the position of the scatterers within the junction, not the energy level, is essential. This contrasts with the conductance peaks near the pinch-off where G is well below $e^2/3h$; they shift diagonally with the asymmetric biasing, which is the behaviour expected for resonant levels (see Supplementary Fig. 3b). Thus, we conclude that the conductance oscillations around $G = e^2/3h$ are not caused by the resonant tunnelling but by the Andreev processes in the multiple-scatterer system.

Supplementary Note 5: Magnetic field dependence

We examined the B dependence of the pinch-off characteristics of the 1/3-1 junction. Supplementary Fig. 4a shows a colour plot of G in the V_S-B plane. The conductance oscillations with $G > e^2/3h$ are observed over the measured range of 8.8 T < B < 9.2 T, indicating the Andreev processes remain present with a slight change in B. Near $V_S = -1.113$ V, G seems to oscillate as a function of B showing $G > e^2/3h$ at several peaks (Supplementary Fig. 4b). The oscillating B dependence may be related to the Aharonov-Bohm interference between tunnelling amplitudes through different scatterers.

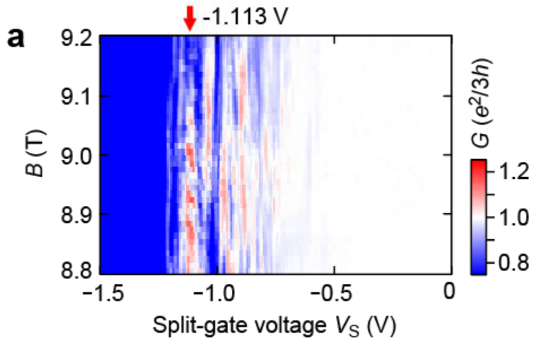

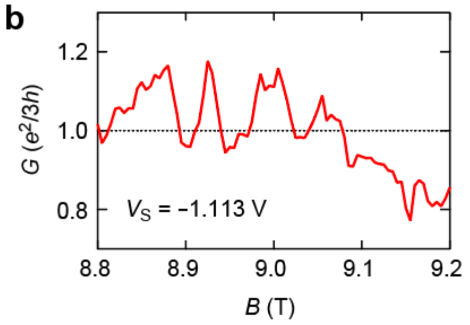

Supplementary Figure 4. a, Colour plot of G as a function of V_S and B. **b,** B dependence of G at $V_S = -1.113$ V.

Supplementary Note 6: Different samples

The signature of the Andreev process, $G > e^2/3h$, is observed not only in the split-gate device demonstrated in the main text, which has the split-gate opening of 300 nm, but also in other samples having wider openings. We examined two samples, one with 600 and the other with 900-nm openings, fabricated on the same wafer. Supplementary Fig. 5 shows the pinch-off characteristics of these samples. Like the 300-nm device, both of them show the conductance oscillations with $G > e^2/3h$, manifesting the Andreev reflection. It is worth noting that their oscillation amplitudes are smaller than those for the 300-nm device, suggesting enhanced equilibration or weaker couplings due to strong negative V_S in the wider samples.

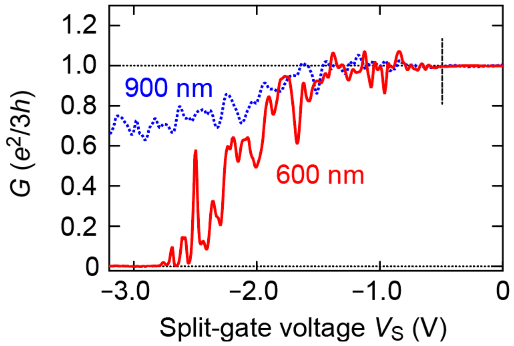

Supplementary Figure 5. Signatures of the Andreev reflection in different samples.

Supplementary Note 7: Transport properties of different quantum Hall junctions

It is instructive to look at the transport properties of other QH junctions having different sets of filling factors. Supplementary Fig. 6a shows the pinch-off characteristics of a junction between $\nu = 1/3$ states formed by applying the top-gate voltages $V_L = V_R = -0.42$ V at $V_{BG} = 1.29$ V and $B = 9$ T. Note that G never exceeds the conductance $e^2/3h$ over the entire range of V_S. At $V_S > -0.55$ V, where the 2DES under the split gate is not depleted, we observe $G < e^2/3h$, which results from the enhanced backscattering through puddles of different filling factors. Below $V_S = -0.55$ V, the conductance through the narrow junction is maintained at $G \cong e^2/3h$ down to $V_S = -0.95$ V. Below -0.95 V, it decreases to zero, indicating that the junction is completely pinched off at $V_S \cong -1.2$ V. Supplementary Fig. 6b displays another result obtained from an IQH junction between $\nu = 1$ and $\nu = 3$ states. The system was prepared by applying $V_L = -0.42$ V at $V_{BG} = 1.29$ V and $B = 3$ T. Likewise, in this case, G decreases from e^2/h to zero without showing $G > e^2/h$ over the entire range of V_S. Thus, the signature of Andreev reflection is neither observed for a junction between the same $\nu = 1/3$ states nor between different IQH states. This, in turn, clearly exhibits that the conductance oscillations with $G > e^2/3h$, demonstrated in the main text, are responsible for the Andreev reflection at the 1/3-1 junction.

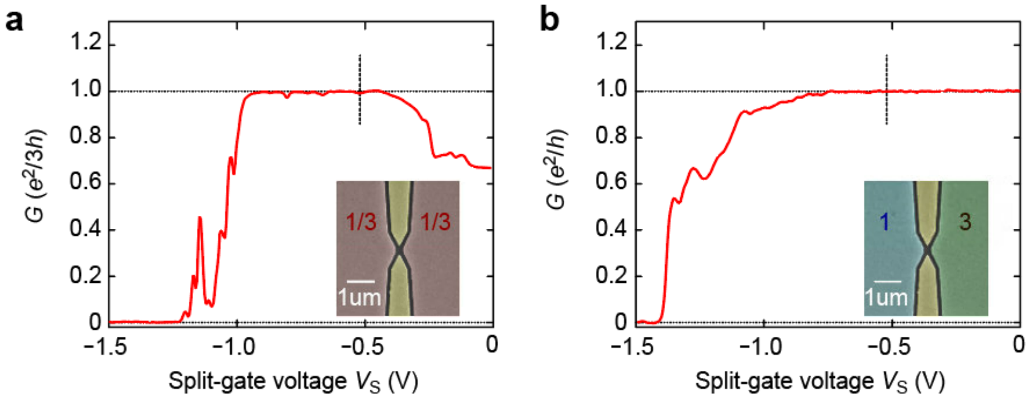

Supplementary Figure 6. a, V_S dependence of G of junction between $\nu = 1/3$ states. **b,** V_S dependence of G of junction between $\nu = 1$ and $\nu = 3$ states.

Supplementary Note 8: Theoretical discussion

The tunnelling problem through a single scatterer between $\nu = 1/3$ and $\nu = 1$ edge states has been studied and solved in the literature[2]. By using a Luttinger liquid representation for the edge states, one can show that this system can be mapped on a junction between two $\nu = 1/2$ edge states, which can be solved exactly using a refermionisation procedure. The total current through the junction, for a bias voltage $V_{\rm in}$ and temperature T, can be written as

$$I(V_{\rm in}, T, T_{\rm k}) = \frac{e^2}{2h} V_{\rm in} - \frac{e}{4h} T_{\rm k} \sinh\left(\frac{V_{\rm in}}{2T}\right) F_3\left(\frac{T_{\rm k}}{2T}, \frac{eV_{\rm in}}{2T}\right), \quad (1)$$

where $T_{\rm k}$ parametrises the coupling strength of the scatterer, and

$$F_3(a,b) = \frac{\pi}{\cosh(ia+b)\cosh(ia-b)} - \frac{i}{\sinh(2b)}\left[\psi\left(\frac{1}{2} - \frac{a+ib}{\pi}\right) + \psi\left(\frac{1}{2} + \frac{a+ib}{\pi}\right) - \psi\left(\frac{1}{2} - \frac{a-ib}{\pi}\right) - \psi\left(\frac{1}{2} + \frac{a-ib}{\pi}\right)\right], \quad (2)$$

with ψ the digamma function.

The most important result is that the conductance of the junction is $e^2/2h$ in the strong-coupling limit (corresponding to $T_{\rm k} \to 0$), and the effective quasiparticles in the transport process are collective excitations with a charge $e/2$, which is different from the charge of the individual excitations existing at each edge. The fact that the conductance reaches $e^2/2h$ means that the output voltage on the $\nu = 1/3$ can be larger than the input one and can be understood in terms of Andreev reflection.

When the junction between the two edge states is wide, it cannot be modelled as a single scatterer, and a model with many scatterers can be used. While the problem becomes much more complicated, one regime where results can be obtained easily is the incoherent regime (we defined "incoherent" in the main text). There, all interference effects are neglected, and the current through a given scatterer depends only on the incoming voltage on the two edges. The incoherent multiple-scatterer model is illustrated in Supplementary Fig. 7 for the case of four scatterers. The voltages $V_1, \ldots V_N$ and $W_1, \ldots W_N$ can be obtained by solving the non-linear system of equations for $n = 1, \ldots N$:

$$V_n = V_{n-1} - \frac{3h}{e^2} I_n\left(V_{n-1} - W_{N-n}, T, T_{{\rm k},n}\right), \quad (3)$$

$$W_n = W_{n-1} + \frac{h}{e^2} I_{N-n+1}\left(V_{N-n} - W_{n-1}, T, T_{{\rm k},N-n+1}\right), \quad (4)$$

where I_n is given by Eq. (1), and V_0 and W_0 are the incoming voltages. One can solve the Eqs. (3) and (4) numerically to obtain the outgoing voltages V_N and W_N, or the output currents from the junction.

When the scatterers are weak, the system can be linearized and solved exactly by going to the continuum limit[3]. For the conductance as a function of the junction width, or the length L of the counter-propagating channels, one obtains

$$G(L,l) = \frac{e^2}{3h} \times \frac{1-\exp(-2L/l)}{1-(1/3)\exp(-2L/l)}, \quad (5)$$

where l is an effective equilibration length, which is related to the coupling strength. This result shows that, in the regime of many weak scatterers, the conductance never goes above $e^2/3h$, and it reaches this value on a length scale $\sim l$. This is the behaviour shown in Fig. 4a with the blue diamonds obtained from the numerical calculation ($T_{\rm k}$ = 36 mK). It is also the behaviour observed

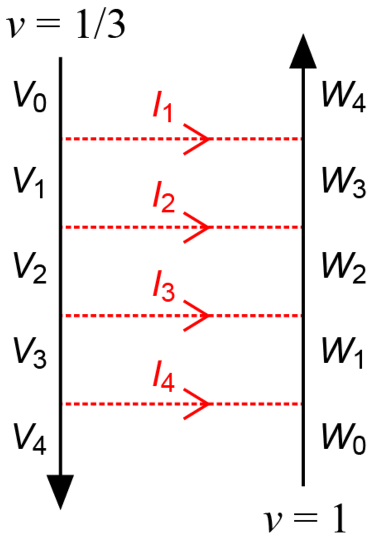

Supplementary Figure 7. Multiple-scatterer model, shown for the case of $N = 4$ scatterers.

experimentally, for the conductance as a function of the split-gate voltage at high V_{in} or T (where oscillations are washed out), in Figs. 3b and 3d. A very different regime is obtained in the strong-coupling limit, where $T_{\text{k},n}$ go to zero to give $g_n = 1/2$ for all n. There, G becomes $e^2/2h$ for an odd N and 0 for an even N, as shown by the black open circles in Fig. 4a. Between these regimes, for scatterers strong enough, a typical behaviour is shown by the red filled circles in Fig. 4a ($T_{\text{k}} = 1.5$ mK), namely, as a function of N, the conductance G oscillates around the value $e^2/3h$, with $G > e^2/3h$ for odd N, and $G < e^2/3h$ for even N. The amplitude of these oscillations decreases as N increases, and G reaches eventually $e^2/3h$ for large N.

In the experiment, one does not have direct access to the number of scatterers. However, the number of scatterers should be roughly proportional to the junction width, which is controlled by the applied split-gate voltage V_{S}. We model the continuous V_{S} dependence of the conductance by making the following reasonable assumptions:

- When no split-gate voltage is applied, the junction consists of a large number N, randomly placed inside the junction. In practice, we put 40 scatterers, with equal strength T_{k}, inside the range [−10,10] on the x-axis.
- The effect of V_{S} is modelled as a window function which reduces the effective width of the junction, with the strength of each scatterer going smoothly from T_{k} to ∞ (= zero-strength scatterer) when the scatterer position goes from inside to outside the effective width determined by V_{S}. In practice, the strength of a given scatterer at position x is provided by the following function of V_{S}:

$$T_k(V_s) = \frac{T_k}{[1-f(x-V_s)]f(x-V_s)}, \qquad (6)$$

where f is a sigmoid function

$$f(y) = \frac{1}{1+\exp(-y/w)}, \qquad (7)$$

where w defines the width of the transition region of the window function (in practice, we have chosen $w = 0.2$). With this choice of window function, all scatterers are suppressed for negative V_S (corresponding to completely pinched-off junction), and the number of active scatterers increases as V_S increases, with all scatterers fully active when $V_S > 10$.

Choosing a relatively strong coupling strength $T_k = 1.1$ mK, with an applied voltage 20 μV at temperature 9 mK (for a single scatterer, these parameters give the conductance ~ 0.49 e^2/h), we obtain the curves in Fig. 4b, each curve corresponding to a different random realization of the position of the scatterers. One can see that the major qualitative features of the experimental results are present; the amplitude of conductance oscillations around $e^2/3h$ decreases as V_S is increased, while the inherent oscillating pattern depends on the fine details of the positions of the scatterers.

Reference

[1] Baer, S., Rössler, C., de Wiljes, E. C., Ardelt, P.-L., Ihn, T., Ensslin, K., Reichl, C., & Wegscheider, W. Interplay of fractional quantum Hall states and localization in quantum point contacts. *Phys. Rev. B* **89**, 085424-1-14 (2014).

[2] Sandler, N. P., Chamon, C. C. & Fradkin, E. Noise measurements and fractional charge in fractional quantum Hall liquids. *Phys. Rev. B* **59**, 12521-12536 (1999).

[3] Nosiglia, C., Park, J., Rosenow, B., & Gefen, Y. Incoherent transport on the $\nu = 2/3$ quantum Hall edge. *Phys. Rev. B* **98**, 115408-1-24 (2018).
